# Attitude towards diabetes and social and family support among type 2 diabetes patients attending a tertiary-care hospital in Bangladesh: a cross-sectional study

**DOI:** 10.1186/s13104-016-2081-8

**Published:** 2016-05-26

**Authors:** Md. Shajedur Rahman Shawon, Fariha Binte Hossain, Gourab Adhikary, Rajat Das Gupta, Mohammad Rashidul Hashan, Md. Fazla Rabbi, G. U. Ahsan

**Affiliations:** Department of Public Health, North South University, Bashundhara, Dhaka, 1229 Bangladesh; Dhaka Medical College, Secretariate Rd, Dhaka, 1000 Bangladesh

**Keywords:** Type 2 diabetes mellitus, Glycemic status, Bangladesh, Social and family support, Attitude, Fasting blood sugar

## Abstract

**Background:**

Bangladesh has been suffering from an epidemiological transition from infectious and maternal diseases to non-communicable lifestyle-related diseases like diabetes, cardiovascular diseases, cancers etc. The burden of diabetes has been increasing rapidly due to high incidence as well as poor glycemic control leading to various macro and micro-vascular complications. In this study, we aim to assess the attitude towards diabetes and social and family support among the Bangladeshi type 2 diabetic mellitus (T2DM) patients.

**Methods:**

This was a cross-sectional study among 144 patients with T2DM at the medicine outpatient department of Dhaka Medical College Hospital (DMCH) in Dhaka, Bangladesh between 1 July and 31 July 2014. Data collection was done by interviewing patients using structured questionnaire. Understanding diabetes, education/advice received, attitude towards diabetes, family and friend support were measured by validated scales adapted from diabetes care profile.

**Results:**

This study includes a total of 144 patients (101 males and 43 females) with type 2 diabetes aged between 20 and 84 years. 87 % of the patients had inadequate blood glucose control (fasting blood sugar >7.2 mmol/L or >130 mg/dl). Statistically significant differences were observed in the mean scores of various attitude scales (i.e. positive, negative, care ability and self-care adherence scale) among patients with adequate and inadequate blood glucose control (p < 0.05). Statistically significant positive correlations were found between these three categories of social and family support. Self-satisfaction with diabetic care was significantly associated with adequate blood glucose control (p = 0.05).

**Conclusions:**

Positive attitude towards diabetes management and support from friends and family were associated with adequate diabetes management. Appropriate public health interventions should be designed to educate and motivate the family members to offer greater support to the diabetes patients.

## Background

Type 2 diabetes mellitus (T2DM) is a growing clinical and public health problem globally. International Diabetes Federation (IDF) has reported that T2DM is affecting approximately 171 million people worldwide and 80 % of all people affected by diabetes are from the low and middle income countries (LMICs) [1–3]. South East Asia would most likely to experience the highest toll from this epidemic as diabetes prevalence is expected to rise by 71 % within next 25 years and countries in this region are least equipped to tackle this emerging crisis [2–4].

There has been an emerging epidemic of non-communicable lifestyle diseases like diabetes, cardiovascular diseases, cancer etc. in Bangladesh. This epidemiological transition from infectious and maternal diseases to chronic diseases is mainly due to demographic transition, increased prevalence of obesity and adoption of westernized lifestyles [5]. According to IDF report (2011), the number of diabetes patients in Bangladesh is approximately 8.4 million which is going to be doubled by the year 2030 [6]. According to a recent meta-analysis, the pooled prevalence of type 2 diabetes mellitus was 6.7 % (4.9–8.6 %) [7]. Higher prevalence of diabetes was observed in urban (8.1 %) compared to rural (2.3 %) population [4]. Another study found the prevalence of T2DM was 5 % in middle-income neighborhood in the capital city, Dhaka [8].

T2DM patients suffer from various micro and macrovascular complications due to inadequate glycemic control. These complications add up to the public health burden of T2DM [9]. Good glycemic control leads not only to higher quality of life [10], but also reduces long-term complications [11, 12]. However, previous studies showed high proportions of diabetes patients had inadequate glycemic control [13, 14].

Although better management of T2DM depends on many factors [15–19], patient’s attitude towards diabetes along with social and family support contribute to better management of diabetes [20]. Diabetes patients can obtain support towards their disease from family members, friends, healthcare providers and patient networks which can lead to better control of diabetes [21–24]. Conversely, lack of social and family support reduce the motivation and effort towards self-care management of diabetes [25].

In this study, we aim to assess the attitude towards diabetes and social and family support among the Bangladeshi type 2 diabetic patients. The result of this study will guide the clinicians and public health policy makers to develop and implement diabetes control programs in Bangladesh and other developing countries.

## Methods

### Study design and population

A cross-sectional study was carried out among 144 patients with type 2 diabetes at the medicine outpatient department of Dhaka Medical College Hospital (DMCH) in Dhaka, Bangladesh between 1 July and 31 July 2014. DMCH is the largest public hospital providing affordable healthcare to a large number of patients from all socio-economic strata through its outpatient, inpatient and emergency facilities [26].

All consecutive adult patients aged 18 years or more who had been previously diagnosed with type 2 diabetes by a qualified healthcare professional e.g. physician, nurse, SACMO (Sub-assistant Community Medical Officer) were included in the study. The exclusion criteria for this study were—(i) Patients with type 1 diabetes mellitus; (ii) patients with gestational diabetes; (iii) Newly diagnosed with type 2 diabetes on the day of survey; (iv) Patients with any diabetes-related-complications requiring hospital admission.

### Data collection tool

Data collection was done by face-to-face interview using a structured questionnaire. Information on socio-demographic status, diabetes care, understanding diabetes, education/advice received, family and friend support and self-perception about glycemic control was collected. All available last readings of glycated hemoglobin (HbA1c) and fasting blood sugar measurements were abstracted from patients’ medical records.

Understanding diabetes, education/advice received, attitude towards diabetes, family and friend support were measured by validated scales adapted from diabetes care profile (DCP) [27]. DCP was developed by Michigan Diabetes Research and Training Center to measure the social and psychological factors associated with diabetes [28].

Attitude towards diabetes scale consisted of 17 items and measured positive attitude, negative attitude, care ability, importance of care, and self-care adherence. Each item was rated on a five-point Likert scale (1 = strongly disagree, 2 = disagree, 3 = neutral, 4 = agree, and 5 = strongly agree). Family and friend support for diabetes questions were divided into 3 categories—support needed, support received and support attitude. In each category there were six items with five-point likert scale (1 = strongly disagree, 2 = disagree, 3 = neutral, 4 = agree, and 5 = strongly agree). Reverse scoring was done for negatively worded questions. Mean scores were calculated for each category by summing the scores of respective items and then divided by count of non-missing items.

Self-perception of glycemic control was assessed by using a scale with five levels: very good, good, fair, poor and very poor. A single global question “If you were to spend the rest of your life with your diabetes treatment the way it is today, how you would feel about this? (very satisfied, somewhat satisfied, neither dissatisfied nor satisfied, somewhat dissatisfied, or very dissatisfied”) was asked to measure satisfaction with current diabetes treatment [29].

A draft questionnaire was developed and piloted on a sample of volunteer patients to refine the wording of the items and ensure clarity of text. The questionnaire was translated into Bengali and then, again translated back to English to maintain the consistency in the translation process. Three qualified research physicians were involved in data collection.

### Operational definitions

Patients were considered to have type 2 diabetes if they were diagnosed by a qualified healthcare professional e.g. physician, nurse, SACMO etc. Patients who responded “Strongly agree” or “Agree” to the question “*Do you follow the diet plan advised by your physician/dietitian?”* were categorized as adherent to diet. Patients were considered as adherent to treatment plan if they responded “Strongly agree” or “Agree” to the question “*Do you follow the treatment plan advised by your physician?*” Physically active was considered if patients were engaged in doing exercise at least for 30 min for three or more days in a week. Diabetes treatment modality was categorized into oral anti-hyperglycemic agent (OAA), alone, insulin alone and oral anti-hyperglycemic agents (OAA) and insulin together. Regular checkup for diabetes was considered if the patients said they visited doctor for diabetes treatment at least once in 2 months or more frequently. We defined inadequate blood glucose control if fasting blood glucose ≥7.2 mmol/L (or 130 mg/dl). Patients were categorized as satisfied with current diabetic treatment if they answered either “very satisfied” or “somewhat satisfied” to the question “*If you were to spend the rest of your life with your diabetes treatment the way it is today, how you would feel about this?*”

### Statistical analysis

Data were presented as frequency and proportions for categorical variables and mean ± standard deviation (SD) for continuous variables. Appropriate statistical tests i.e. Chi square tests, independent sample t tests were performed. Correlation matrix was developed to find out the association between support needed, support received and support attitude. All statistical tests were considered significant at *p* value <0.05. Statistical analyses were performed using SPSS version 22.0 for Windows (IBM, NY, USA).

## Results

### Participants’ characteristics

This study included a total of 144 patients (101 males and 43 females) with type 2 diabetes aged between 20 and 84 years. Majority of the participants were married (78.5 %) and followed Islam (86.1 %) as religion. 29.2 % of the participants were working fulltime (35 h or more in a week); 23.6 % were homemakers; about 27 % were either retired or unemployed and not looking for work. Among the participants 54 % were non-smoker, 27 % were current smoker and 19 % were ever smoker. More than half (61.8 %) of the participants had concurrent history of hypertension and about one-third had history of dyslipidemia. The socio-demographic and clinical characteristics of the participants is given below [see Table 1].

**Table 1:** Socio-demographic and clinical characteristics of the participants (n = 144)

Variables	Categories	Number (%)
Gender	Male	101 (70.1 %)
	Female	43 (29.9 %)
Age in years	Mean ± SD	54.4 ± 11.7
Marital status	Married	113 (78.5 %)
	Widowed	21 (14.6 %)
	Separated/divorced	05 (3.5 %)
	Never married	05 (3.5 %)
Religion	Islam	124 (86.1 %)
	Hindu	19 (13.2 %)
	Others	01 (0.7 %)
Employment status	Working full time	42 (29.2 %)
	Working part-time	17 (11.8 %)
	Unemployed or laid off and looking for work	07 (4.9 %)
	Unemployed and not looking for work	18 (12.5 %)
	Homemaker	34 (23.6 %)
	Retired	21 (14.6 %)
	Others	05 (3.5 %)
Smoking status	Non-smoker	78 (54.2 %)
	Current smoker	39 (27.1 %)
	Ever smoker	27 (18.8 %)
Concurrent history of hypertension	Yes	89 (61.8 %)
	No	30 (20.8 %)
	Don’t know	25 (17.4 %)
Concurrent history of dyslipidemia	Yes	48 (33.3 %)
	No	35 (24.3 %)
	Don’t know	61 (42.4 %)

### Information about diabetes

The mean age of diagnosis with type 2 diabetes was 45.62 ± 10 years and mean duration of suffering was 8.9 ± 7.1 years. Half of the respondents had family history of diabetes. Majority of the patients (42 %) used only OAA, 30 % used only insulin and 28.5 % used both OAA and insulin as their treatment modalities. 50 % patients were having regular checkup (once in 2 months or more frequently) by physician for diabetes. Majority of the patients reported that they followed the advised treatment plan (79.9 %), diet plan (79.9 %) and were physically active (50.3 %) [see Table 2].

**Table 2:** Information related to diabetes and diabetic care among the participants

Variables	Categories	Number (%)
Age of diabetes diagnosis, in years	Mean ± SD	45.62 ± 10
Duration of diabetes, in years	Mean ± SD	8.9 ± 7.1
Family history of diabetes	Yes	73 (50.7 %)
	No	35 (24.3 %)
	Don’t know	36 (25 %)
Treatment modalities	OAA only	60 (41.7 %)
	Insulin only	43 (29.9 %)
	OAA and insulin	41 (28.5 %)
Regular checkup for diabetes	Yes	72 (50 %)
	No	72 (50 %)
Following treatment plan advised by the physician	Yes	115 (79.9 %)
	No	29 (20.1 %)
Following diet plan advised by physician/dietitian	Yes	101 (70.1 %)
	No	43 (29.9 %)
Physically active	Yes	72 (50.3 %)
	No	71 (49.7 %)
Suffered from any diabetes related complications	Yes	89 (61.8 %)
	No	55 (38.2 %)
Testing blood sugar level regularly	Yes	114 (79.2 %)
	No	30 (20.8 %)
Keeping record of blood sugar test results	Yes	44 (30.6 %)
	No	56 (38.9 %)
	Only unusual values	44 (30.6 %)
Availability of HbA1C information	Yes	80 (55.6 %)
	No	64 (44.4 %)
Availability of fasting blood sugar information	Yes	132 (91.7 %)
	No	12 (8.3 %)

*OAA* oral anti-hyperglycemic agents; *HbA1c* glycated hemoglobin

Most of the patients (80 %) reported that they regularly measured their blood sugar level and we found fasting blood sugar information for the last month among 92 % patients in their record books. On the other hand, only 55.6 % patients had HbA1C information for the last 3 months. Due to lack of HbA1C information in many participants, we calculated adequate blood glucose control (>7.2 mmol/L or >130 mg/dl) and found only 13 % patients had adequate control.

### Attitudes towards diabetes

**Fig. 1:**
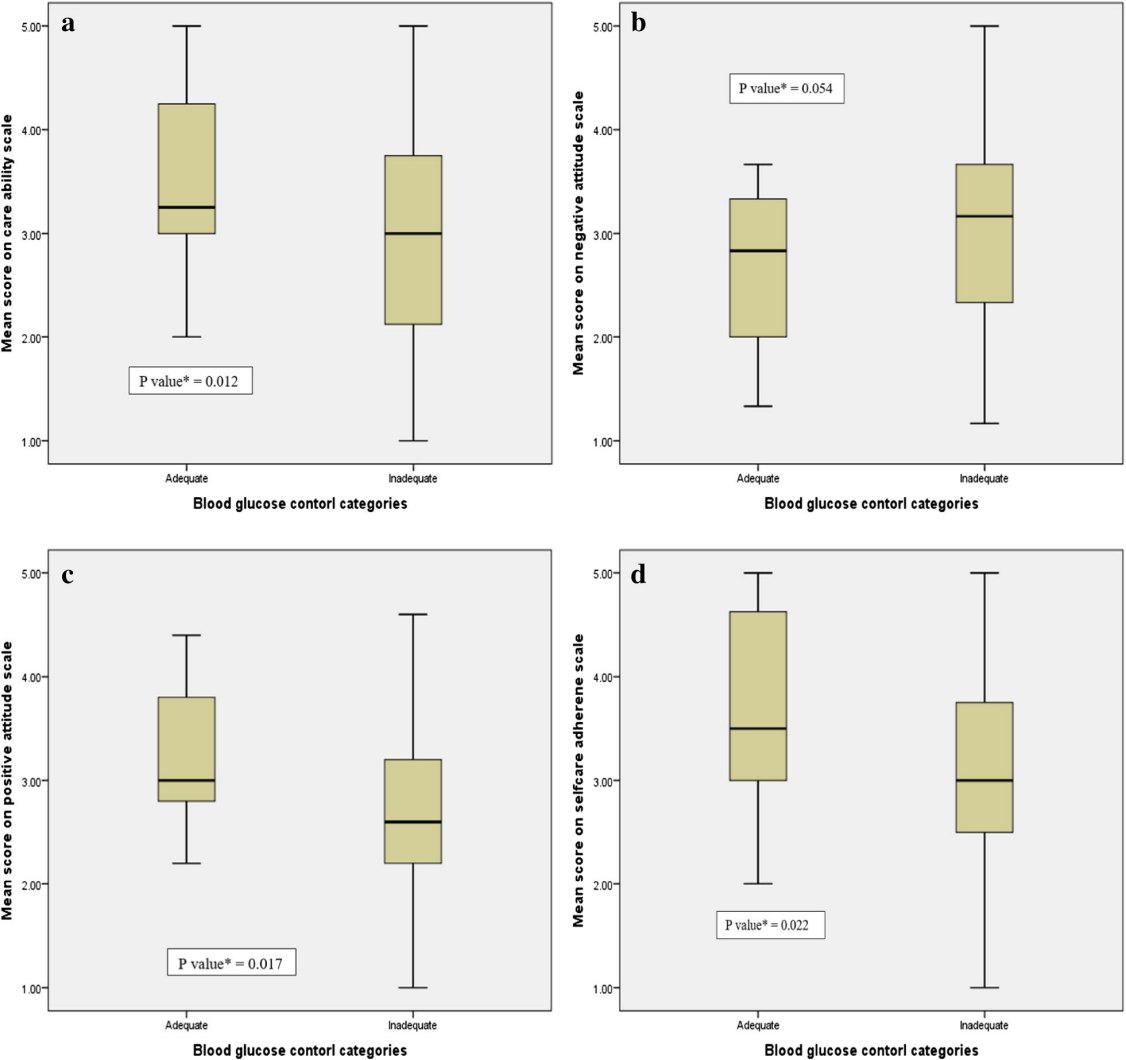
*Box-plots* showing distribution of mean scores of **a** positive attitude towards diabetes, **b** negative attitude towards diabetes, **c** attitudes towards diabetic care ability and **d** attitudes towards diabetic self-care adherence by blood glucose control categories among the participants

Attitude towards diabetes was analyzed into five different dimensions i.e. positive attitude, negative attitude, care ability, importance of care and self-care adherence. Statistically significant differences were observed in the mean score of positive attitude scale, negative attitude scale, care ability scale and self-care adherence scale among patients with adequate and inadequate blood glucose control (see Fig. 1).

### Social and family support

**Fig. 2:**
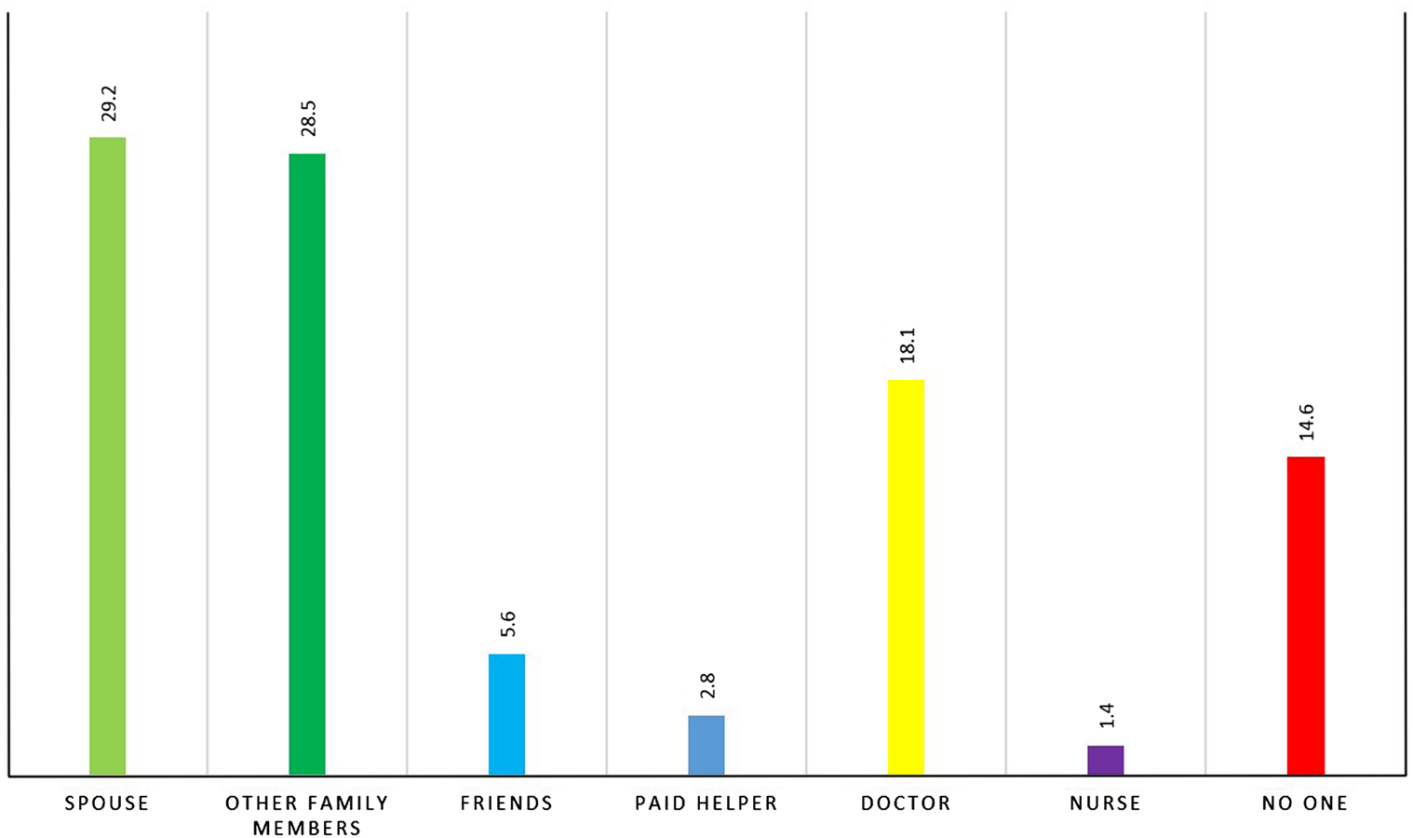
Most caring person helping with diabetes self-care management among the participants (n = 144)

Patients with adequate blood glucose control had higher mean scores than patients with inadequate blood glucose control on support needed scale (4.2 vs. 3.8; p = 0.160), support received scale (3.7 vs. 3.2; p = 0.056) and support attitude (4.1 vs. 3.7; p = 0.067). Statistically significant positive correlations were found between these three categories of social and family support (see Table 3). Spouse (29 %) and family members (28 %) are found to be the care-giver for most of the diabetic patients. Approximately 14 % of the patients said that they had no caregiver for their diabetes (see Fig. 2).

**Table 3:** Correlation matrix for support needed, support received and support attitude among the participants

	Support needed	Support received	Support attitude
Support needed	1	0.269^a^	0.193^b^
Support received	0.269^a^	1	0.675^a^
Support attitude	0.193^b^	0.675^a^	1

^a^Pearson correlation is significant at the 0.01 level (2-tailed)

^b^Pearson correlation is significant at the 0.05 level (2-tailed)

### Self-perception and satisfaction about diabetes

Most of the patients had good perception about their glycemic control and only 29 % patients perceived their glycemic control as “poor” or “very poor”. 42 % patients were either “very satisfied” or “somewhat satisfied” if they were to spend the rest of their lives with current diabetes treatment. Self-satisfaction with diabetic care was significantly associated with adequate blood glucose control (p = 0.05).

## Discussion

The noteworthy findings of this study are: (i) Type 2 diabetes patients, in general, lack information about HbA1C and other related clinical parameters; (ii) The proportion of T2DM patients with inadequate blood glucose control is 87 % according to fasting blood sugar cut-off ≥7.2 mmol/L or 130 mg/dl; (iii) Significant differences have been observed in the mean scores of attitude between patients with adequate and inadequate blood glucose control; (iv) Significant positive correlation was observed between support attitude of friends and family and support received; (v) Most of the diabetes patients had good perception as well as satisfaction about their diabetes management despite the fact that most of them failed to achieve the recommended level of fasting blood glucose for T2DM patients.

The proportion of T2DM patients with poor management of their diabetes was high (87 %) among the participants, and it was much higher than the findings from studies conducted in many countries. For instance, studies with large sample sizes conducted in the UK [30], Canada [31], Brazil [32] and Venezuela [29] found that the prevalence of inadequate diabetes control were 76, 73, 73 and 75 %, respectively. However, some studies found much lower prevalence of poor diabetes control [33, 34]. Furthermore, in the US, estimates from the National Health and Nutrition Examination Survey (NHANES) showed a decreasing trend in the prevalence of poor diabetes management (i.e. from 63 to 43 % over the period from 1999 to 2004) [35]. In spite of the fact that these variations in prevalence of poor glycemic control across different settings might be true, methodological differences (i.e. selection of study populations, methods/definition of outcome ascertainment) might lead to the differences in prevalence estimates [29].

We found that poor blood glucose control was more common among patients who did not follow the advised diet plan. Therefore, patients should be encouraged to follow the diet plan as prescribed by the physician or dietitian. Despite the importance of physical activity and regular checkup by healthcare professionals in controlling diabetes status, a major portion of the participants was found physically inactive as well as not followed on regular basis (see Table 4). Therefore, public health interventions focusing on educating and motivating T2DM patients on regular physical activity and follow-up are crucial.

**Table 4:** Variables associated with adequate blood glucose control among the type 2 diabetes patients

Variables	Categories	Adequate blood glucose control n (%)	Inadequate blood glucose control n (%)	P value
Age, in years	Mean ± SD	49.7 ± 8.8	54.4 ± 12.1	0.133**
Sex	Male	12 (13.2 %)	79 (86.8 %)	0.875*
	Female	5 (12.2 %)	36 (87.8 %)	
Duration of suffering from diabetes	<5 years	6 (13.3 %)	39 (86.7 %)	0.911*
	≥5 years	11 (12.6 %)	76 (87.4 %)	
Suffered from diabetes related complications	Yes	6 (7.3 %)	76 (92.7 %)	0.015*
	No	11 (22.0 %)	39 (78.0 %)	
Testing blood sugar regularly	Yes	14 (13.2 %)	92 (86.8 %)	0.820*
	No	3 (11.5 %)	23 (88.5 %)	
Regular checkup for diabetes	Yes	12 (17.6 %)	56 (82.4 %)	0.092*
	No	5 (7.8 %)	59 (92.2 %)	
Adherent to diet plan	Yes	17 (18.5 %)	75 (81.5 %)	0.004*
	No	0 (0.0 %)	40 (100.0 %)	
Adherent to treatment plan	Yes	16 (15.2 %)	89 (84.8 %)	0.194***
	No	1 (3.7 %)	26 (96.3 %)	
Physically active	Yes	11 (16.4 %)	56 (83.6 %)	0.231*
	No	6 (9.4 %)	58 (90.6 %)	
Social and family support [mean ± SD]	Score on support needed scale	4.2 ± 0.66	3.8 ± 0.9	0.160**
	Score on support received scale	3.7 ± 0.9	3.2 ± 1.0	0.056**
	Score on support attitude scale	4.1 ± 0.9	3.7 ± 0.9	0.067**
Support received from family and friends	Yes	4 (6.6 %)	57 (93.4 %)	0.032*
	No	13 (19.4 %)	54 (80.6 %)	

* Chi square test; ** Independent sample t test; *** Fisher’s exact test

We observed a significant positive correlation between support attitude and support received from family and friends. Also, mean score on support received was higher in patients with adequate blood glucose control than that of inadequate control group (p = 0.056). Receiving support from friends and family was significantly associated with good control of blood glucose level. A mixed-method study previously found that lack of support from friends and family members led to poor management of diabetes which was mediated by less adherence to treatment [36]. We found, as expected, that most of the patients got highest help for their diabetes self-care management from their spouse or family members (58 %). Therefore, future interventions for diabetes control and prevention should also involve family members and/or primary caregivers in order to improve their motivation and behavioral skills to offer greater support to the diabetes patients.

Only 71 % of the surveyed type 2 diabetes patients had overall good perception about their diabetes management. This finding is very alarming because most of these patients actually had poor blood glucose control. Therefore, patient education about good glycemic management is very crucial in order to achieve and maintain better control over diabetes status to stall further complications. We found association between global satisfaction with current diabetic care and adequate blood glucose control among T2DM patients, similar to another study published previously [29].

To the best of our knowledge, this study is the first study in Bangladesh that draws attention to attitude towards diabetes and social and family support among type 2 diabetes patients. However, we were not able to provide any causal inferences between them from this cross-sectional study. Therefore, prospective study involving large number of T2DM patients is warranted to assess the relationship between attitude towards diabetes and support from friends and family and prognosis of diabetes.

There are several limitations of this study. Convenience sampling of the type 2 diabetes patients attending a tertiary hospital, who might be systematically different from the general population, hence might introduce selection bias in the estimates. However, the prevalence of inadequate blood glucose control among T2DM patients not seeking healthcare might be even higher than that of our study sample. Additionally, the study had smaller sample size and information about diabetes were self-reported. However, these findings add to the body of knowledge of diabetes control and prevention of diabetes-related complications among T2DM patients.

## Conclusions

In conclusion, our study found very high proportions of inadequate blood glucose control among T2DM patients. This might contribute to increased incidence of various macro-vascular and micro-vascular diabetic complications and incur huge economic burden on the healthcare system. Positive attitude towards diabetes management and support from friends and family were associated with adequate diabetes management. Such knowledge will aid the health professionals and policy makers to develop and ensure good quality diabetic care and health education. Moreover, appropriate public health interventions should be designed to educate and motivate the family members to offer greater support to the diabetes patients.

## Abbreviations

DCP: diabetes care profile
DMCH: Dhaka Medical College Hospital
HbA1c: glycated hemoglobin
IDF: International Diabetes Federation
LMICs: low and middle income countries
OAA: oral anti-hyperglycemic agents
SACMO: sub-assistant community medical officer
SD: standard deviation
T2DM: type 2 diabetes mellitus
